# Porous Si-Based Materials for Lithium-Ion Battery Anodes: Structural Design and In Situ/Operando Characterization

**DOI:** 10.3390/ma19030582

**Published:** 2026-02-02

**Authors:** Yiming Zhang, Chang Luo, Xijun Liu, Zhifeng Wang

**Affiliations:** 1 “The Belt and Road Initiative” Advanced Materials International Joint Research Center of Hebei Province, School of Materials Science and Engineering, Hebei University of Technology, Tianjin 300401, China; 2 Arizona College of Technology at Hebei University of Technology, Tianjin 300401, China; 3 School of Resources, Environment and Materials, Guangxi University, Nanning 530004, China

**Keywords:** Li-ion battery, silicon anode, porous structure, in situ/operando characterization

## Abstract

Silicon is a well-known anode material for lithium-ion batteries that has attracted a lot of interests because of its high theoretical specific capacity (4200 mAh g^−1^). However, its severe volume expansion during cycling leads to structural degradation and rapid capacity fading. The design of porous silicon architectures has emerged as a fundamental and effective strategy to mitigate these issues by accommodating mechanical stress and preserving electrode integrity. Concurrently, the development of advanced in situ/operando characterization techniques has shifted the research paradigm, enabling direct observation of dynamic structural and interfacial evolution under operating conditions. This review systematically summarizes recent progress in the rational design of porous Si-based anodes and critically examines how state-of-the-art in situ methods provide direct mechanistic validation of these designs. The work highlights the synergistic interplay between targeted material engineering and in situ/operando characterization, offering a roadmap for the development of high-performance porous silicon anodes.

## 1. Introduction

**Figure 1:**
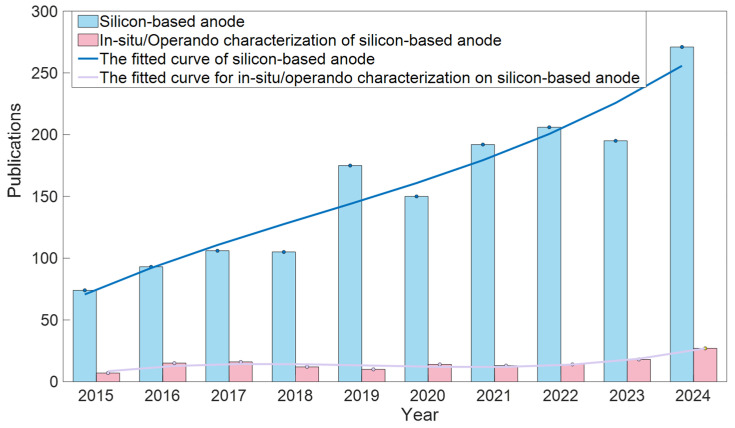
The trend in the research popularity of silicon-based anodes.

The 21st century has witnessed a rapid transformation towards new energy technologies worldwide. Lithium-ion batteries (LIBs) have become the core cornerstone in this transformation due to their high energy density and excellent cycling stability. LIBs, as the most widely used power source in portable electronic devices, electric vehicles, and other fields, continue to attract a large amount of research attention from the global scientific community. With the increasing demand for energy storage in application scenarios, researchers have been continuously pursuing higher-energy-density batteries, which has driven them to explore alternative anode materials beyond commercial graphite anodes 372 (mAh g^−1^) [1,2,3,4,5,6]. To date, researchers have discovered multiple potential candidate materials, including silicon (Si), phosphorus (P), tin (Sn), and their composite materials, all of which have theoretical specific capacities far exceeding that of graphite [7,8]. Among numerous alternative materials, silicon has become one of the most promising anode materials due to its unparalleled high theoretical capacity (corresponding to the Li_22_Si_5_ phase, 4200 mAh g^−1^), suitable working potential, and abundant natural reserves. However, in order to achieve practical applications of this highly promising silicon anode, a key challenge still needs to be overcome. During lithiation and delithiation processes, the volume expansion rate of silicon can reach as high as 300–400%, and this significant volume change can cause significant mechanical stress, leading to electrode cracking, silicon particle breakage, and loss of electrical contact between the active material and the collector. These issues will directly lead to a decrease in electrode conductivity, deterioration of cycling stability, and rapid capacity degradation of the battery [9]. In addition, the repeated fragmentation of silicon particles can lead to the continuous rupture and regeneration of the solid–electrolyte interface (SEI) layer, which excessively consumes lithium ions and electrolytes, further threatening the lifespan of the battery. To overcome the above challenges, researchers have explored various technical strategies [10,11,12,13,14,15,16]. For example, Huo et al. elucidated the failure mechanism of silicon anodes in solid-state batteries [17]. Yang et al. proposed a novel transfer-printing pretreatment method that effectively improves the structural integrity of silicon electrodes [18]. In recent years, the global research attention on silicon-based anodes has continued to rise (Figure 1), and the number of published studies has been increasing year by year. This surge in research activities not only highlights the urgent need to develop high-performance silicon-based anodes but also confirms their enormous potential for application in the next generation of high-energy-density batteries.

**Figure 2:**
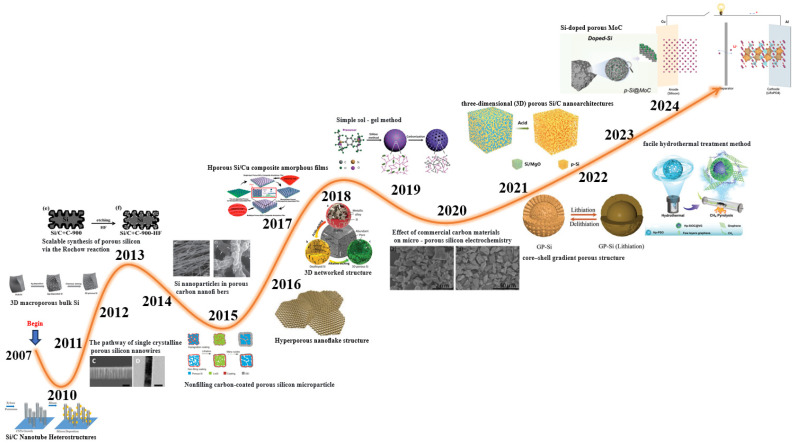
Historical development of porous Si-based materials as anodes for lithium-ion batteries. Reproduced from [22,28,29,31,33,34], with permission from Wiley; Reproduced from [30,32], with permission from Elsevier; Reproduced from [20,21,23,24,25,26], with permission from American Chemical Society.

The structural design of porous silicon has become a transformative strategy for significantly improving the electrochemical performance of silicon-based anodes. The origin of this innovative method can be traced back to the research of Bao et al., who first proposed and prepared microporous silicon architectures [19]. Since the emergence of this breakthrough achievement, the field has entered a period of rapid development, as shown in Figure 2 [20,21,22,23,24,25,26,27,28,29,30,31,32,33,34]. Various multidimensional structural design schemes have been successively developed. To date, researchers have successfully constructed porous silicon in different dimensional configurations such as zero-dimensional (0D), one-dimensional (1D), two-dimensional (2D), and three-dimensional (3D) configurations, each of which has been optimized specifically to eliminate the performance shortcomings of silicon anodes. In addition to regulating dimensions, the optimization of structural design is also a research focus. Researchers have come up with different structural models, like core–shell structures, yolk–eggshell structures, and silicon sphere composite hybrid materials. These detailed designs can ease the volume expansion issue of silicon when going through repeated charge–discharge cycles, keep the electrode structures intact, and boost battery performance. At the nanoscale regulation level, the issue of doping has been demonstrated to significantly improve the conductivity and mechanical stability of porous silicon materials. With the development of preparation technology, the preparation process has become more mature, and the electrochemical performance of porous silicon has improved continually. Despite the significant improvement in research on silicon anodes, a few issues should also be addressed. For example, how can the structure be designed in such a way that it would achieve optimal performance? Which is the best preparation method to use in cases of large-scale commercial production? To overcome these difficulties, it is important to further explain the dynamics of silicon when it is subjected to electrochemical cycles, which can be achieved through the application of sophisticated in situ characterizations methods. This technology is capable of providing insights into the working of silicon anodes in a very intuitive manner that is highly relevant to the design of the next-generation high-performance lithium-ion battery with a long lifetime.

**Figure 3:**
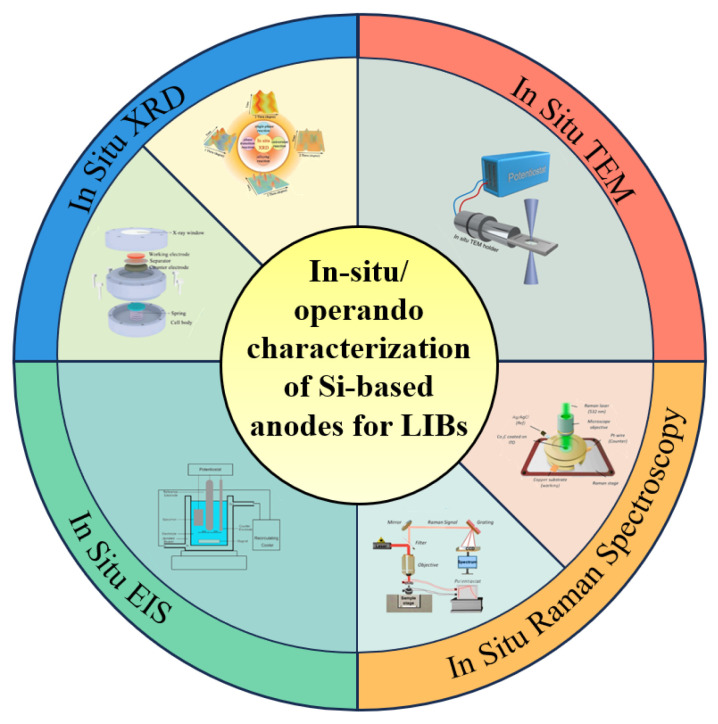
Advanced in situ/operando characterization and detection equipment for Si-based anode studies. Reproduced from [35,39], with permission from Wiley; Reproduced from [40], with permission from Elsevier; Reproduced from [37,38], with permission from American Chemical Society.

With the development of in situ characterization technology, researchers have made it possible to study the failure mechanisms arising during the working of silicon-based anodes, representing a significant shift as compared to the traditional ex situ methods. With advanced technologies, it is possible to monitor electrochemical performance and structural development in real time and with dynamism, introducing completely new perspectives of study. In this situation, in situ X-ray diffraction (XRD) has been demonstrated to be a necessary technical identifying procedure to track the periodical phase changeover that occurs during discharge/charge cycling. In the meantime, in situ transmission electron microscopy (TEM) can enable morphological observation of the in situ changes that occur at the atomic scale in electrochemical processes. In addition, in situ Raman spectroscopy can measure the phase transition and chemical bond evolution within electrode materials, and in situ electrochemical impedance spectroscopy (EIS) can provide important information on interface properties and how the impedance spectrum varies during cycling. These advanced technologies have been critical in the full analysis of the structure and electrochemical actions of silicon-based anodes and their usage. With real-time, high-resolution data collection, researchers can pinpoint fault mechanisms with an unprecedented level of precision and devise targeted strategies to enhance anode performance. Figure 3 [35,36,37,38,39,40] shows the instruments related to the four kinds of advanced in situ characterization techniques. The figure shows their unique abilities in detecting the basic working principle of silicon-based anodes. The continuous advancement of these in situ detection methods has facilitated the development of the next generation of lithium-ion batteries.

Although a large number of reviews has explored the structural design of silicon-based anodes, there is a clear research gap at the intersection of structural design and the verification of the dynamic mechanisms involved under battery operating conditions. The research framework of existing relevant literature often presents two complementary but disconnected states: One type focuses on structural engineering optimization of porous silicon, which comprehensively sorts out the material morphology but often ignores the real-time evolution law and failure mechanism of these structures in the electrochemical cycling process [41,42,43,44,45,46,47,48]. Another type of research focuses on elucidating the principles and methods of various in situ characterization techniques [49,50,51,52,53,54,55,56,57,58] but fails to systematically apply these techniques to analyze the complex “structure–performance” correlation characteristics of porous silicon electrodes. This disconnect in studies ultimately results in a misunderstanding of the intrinsic connection between structural design and the actual functional performance of porous silicon.

**Figure 4:**
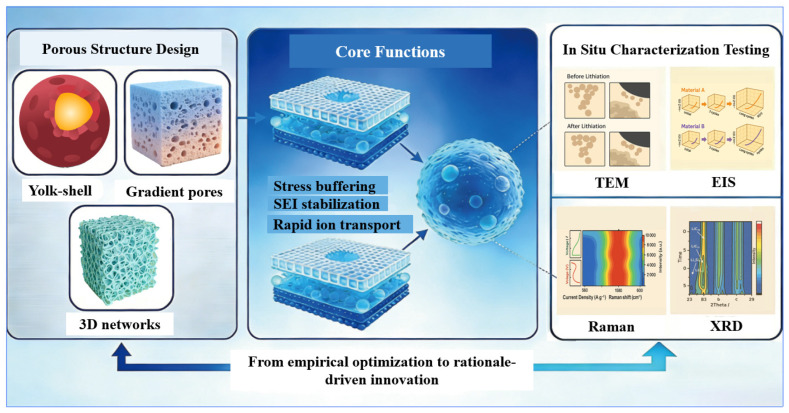
“Design for Validation”: Linking porous architectures, operational functions, and in situ characterization.

To bridge this research gap, this review innovatively proposes a unique “design for validation” framework (Figure 4), with the core goal of achieving the deep integration of the two research dimensions mentioned above rather than simply presenting them side by side. Investigation of porous silicon should not be restricted to only considering its fancy structural design while neglecting the special influences of different structures on its electrochemical performance. On the contrary, when designing a structure, it is necessary to actively utilize the structure to control the electrochemical process and take into account the dynamic changes in the anode materials during actual operation [59]. Therefore, this work systematically links specific porous designs with their expected functions, with the key being to demonstrate how advanced in situ tools can provide direct and time-validated evidence to validate, refine, and even challenge these design principles. By establishing a clear connection from design goals to mechanistic observation, this review provides a forward-looking perspective for the development of high-performance silicon anodes, aiming to guide innovation in this field from empirical optimization to motivation-driven innovation.

## 2. Structural Design of Porous Si-Based Electrodes

### 2.1. Template-Guided Synthesis

**Figure 5:**
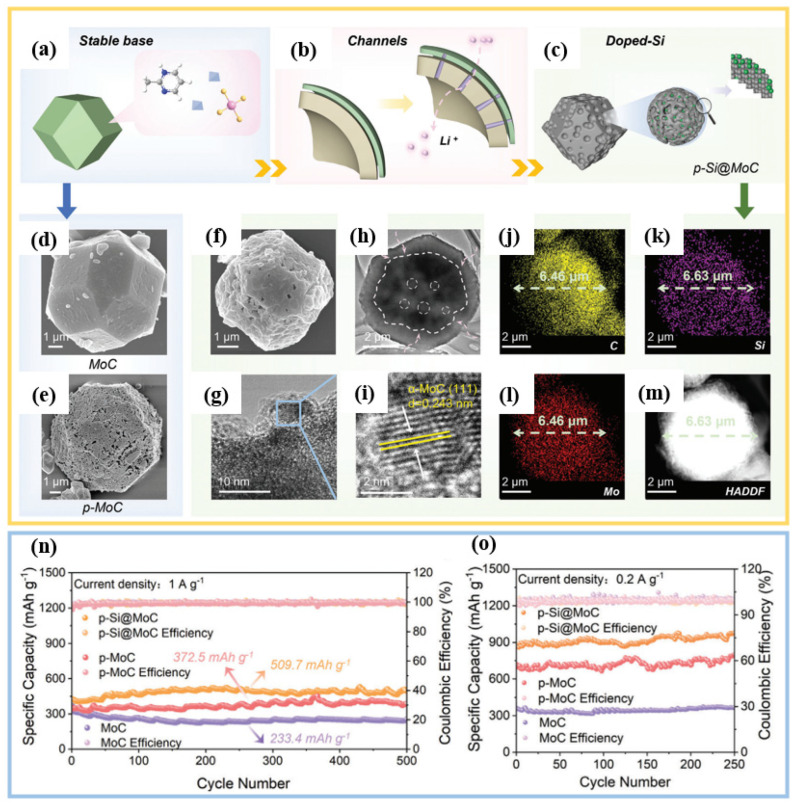
(**a**–**c**) Schematic illustration of the structural design and lithium-ion transport mechanism of SiO_2_@HZIF-ZnMo and p-Si@MoC composite anode materials. (**d**–**f**) A comparative analysis of the SEM images of three anodes. (**g**–**i**) TEM images displaying the p-Si@MoC nanocomposite. (**j**–**m**) Microstructure and elemental distribution of p-Si@MoC. (**n**) Cycling performance of three anodes at a current density of 1 A g^−1^. (**o**) Cycling performance of three anodes at 0.2 A g^−1^. Reproduced from [34], with permission from Wiley.

Chen et al. demonstrated a representative example of template-guided synthesis, fabricating silicon-doped porous molybdenum carbide anodes [34]. Starting from a precursor of a ZnMo bimetallic zeolite pyridine alkoxide framework (ZIF), a porous Si@MoC composite material was obtained through a magnesium thermal reduction process (Figure 5a–m). The as-obtained material retains the multi-faceted morphology of the original ZIF (Figure 5f), while its open-pore structure facilitates efficient Li^+^ transport (Figure 5b). More importantly, the low doping level and uniform dispersion of silicon effectively reduce the mechanical strain caused by repeated volume changes, thereby improving the durability of the electrode (Figure 5c). Structural characterization confirmed that the integrity of the framework was maintained after pyrolysis (Figure 5d,e), and EDS mapping (Figure 5j–m) verified the uniform distribution of elements in the composite material. In terms of electrochemistry, the material has a high specific capacity of 976.6 mAh g^−1^ after 250 cycles at 0.2 A g^−1^, exhibiting excellent cycling stability (Figure 5n,o).

**Figure 6:**
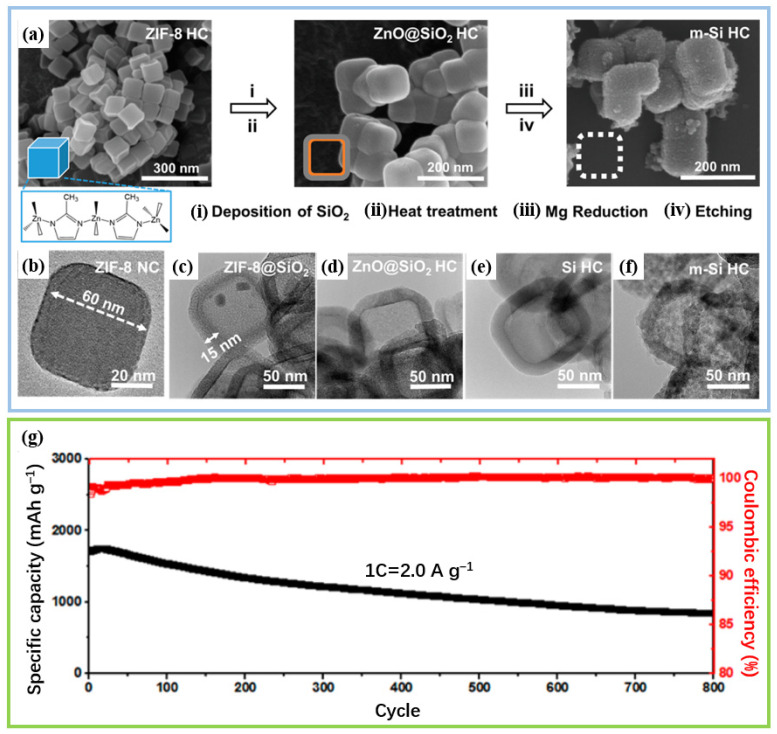
Preparation process, morphology and electrochemical properties of m-Si HC. (**a**) Schematic illustrating the key synthesis steps of m-Si hollow carbon. TEM images tracking the structural evolution: (**b**) ZIF-8 NC; (**c**) ZIF-8@SiO_2_; (**d**) ZnO@SiO_2_ HC. (**e**,**f**) Si hollow nanocubes obtained at different Mg ratios: (**e**) Si HC (1:0.4); (**f**) m-Si HC (1:0.8). (**g**) Cycling stability of m-Si HC at 1C. Reproduced from [60], with permission from American Chemical Society.

Yoon et al. [60] presented another example of precise structural engineering through templating. Mesoporous silicon hollow cubes (mSi HCs) were fabricated by core-shell synthesis, using ZIF8 as the sacrificial metal–organic framework template (Figure 6a). The cubic shape is maintained throughout the entire continuous processing step (Figure 6b–f), forming a well-defined hollow cube with an internal cavity of approximately 60 nm and an external shell (Figure 6f). This custom architecture achieves outstanding electrochemical performance. The mSi HC anode maintains a stable capacity of 850 mAh g^−1^, and the coulombic efficiency reaches 99.8% within 800 cycles at a rate of 1C (Figure 6g).

These studies, as a whole, bring to light the key advantages of the template-guided synthesis method. It is also able to recapitulate the pre-determined template structure with precision, and this enables the production of porous silicon material in which the geometry, morphology and size of the pore can all be regulated. This kind of synthesis technique has achieved both accurate control of porous silicon structures and, in the process, good incorporation of high-capacity silicon material in the presence of substrates that are conductive or mechanically stable through a reasonable selection of templates. It is precisely with respect to this aspect that its significant potential in the design of silicon-based composite anode materials has been pointed out.

### 2.2. Etching-Assisted Fabrication

The etching-guided method provides an efficient and scalable pathway for the preparation of porous silicon. Its core principle involves selectively removing specific components from the solid precursor, thereby forming pore structures inside the material. This method usually uses magnesium silicide (Mg_2_Si) and other precursors, and after acid leaching treatment, a three-dimensional interconnected porous silicon network can be constructed.

**Figure 7:**
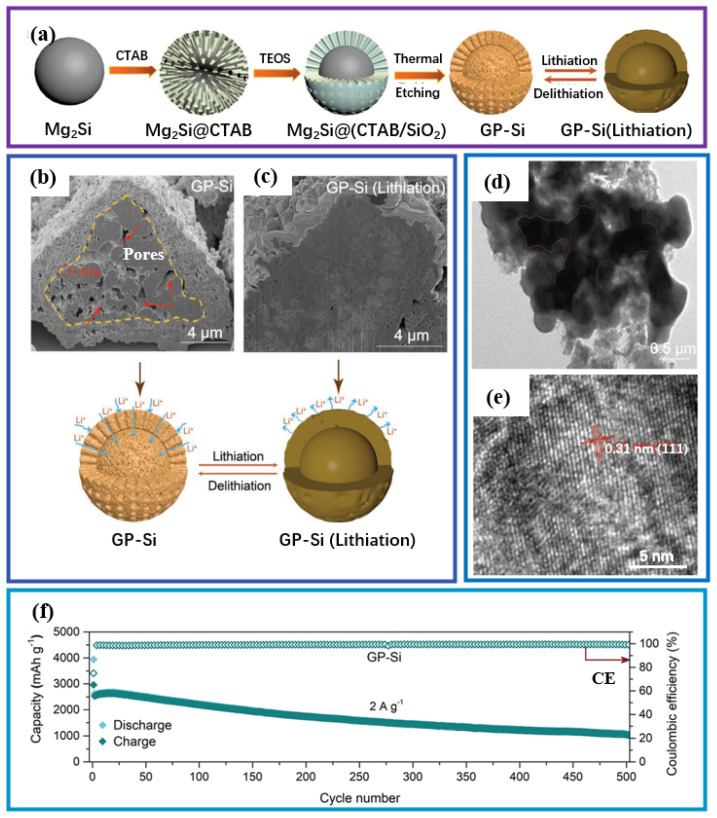
(**a**) Schematic of the GP-Si preparation process. (**b**) FIB-SEM image of pristine GP-Si. (**c**) Image after first discharge to 0.01 V. (**d**,**e**) TEM images of GP-Si. (**f**) Long-term cycling of GP-Si under 2 A g^−1^. Reproduced from [31], with permission from Wiley.

A typical application case of this synthesis strategy is the core–shell-structure gradient porous silicon (GP-Si) reported by Yang et al. [31]. The preparation process is described as follows: A silicon layer is first deposited on the surface of a Mg_2_Si precursor; then, the magnesium oxide generated by the reaction is selectively removed through a controllable etching process. Finally, the target pore structure can be obtained (Figure 7a). GP-Si discloses a unique core–shell structure in which the core is a dense, high-strength layer and the outer shell is a gradient pore structure (Figure 7b–e). This sophisticated design buffers mechanical stress through the progressive compaction resistance provided by the gradient structure, effectively adapting to the significant volume expansion of silicon in electrochemical cycling while also helping to stabilize the solid–electrolyte interface (SEI) film. Electrochemical performance test results (Figure 7f) show that the gradient porous silicon anode exhibits excellent cycling stability. At a current density of 2 A g^−1^, it can still maintain a reversible capacity of 1059 mAh g^−1^ after 500 cycles.

**Figure 8:**
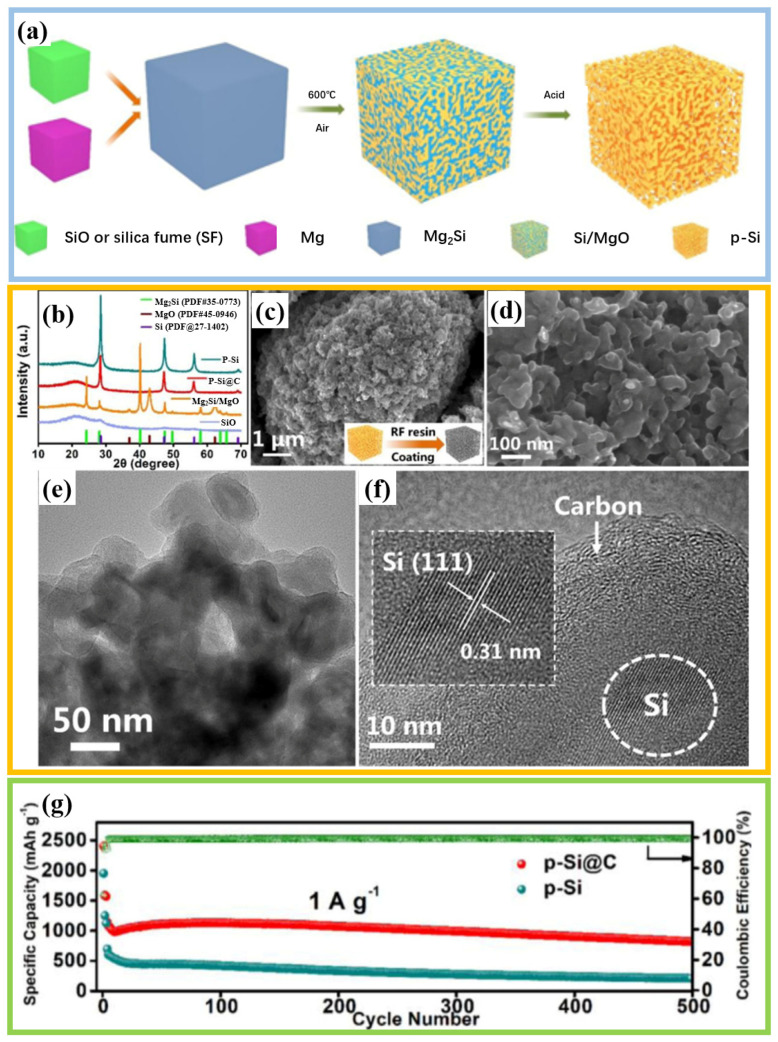
(**a**) Schematic illustration of the design and synthesis process of p-Si. (**b**) Comparison of XRD patterns for different materials. (**c**,**d**) SEM images and illustration of the preparation process of p-Si@C. (**e**) TEM and (**f**) HRTEM images of p-Si@C. (**g**) Cycling performance at 1 A g^−1^. Reproduced from [32], with permission from Elsevier.

The work of Liu et al. further demonstrated the feasibility of constructing an interconnected network through etching strategies [32]. As shown in Figure 8a, the process begins with the preparation of the Mg_2_Si precursor, which is then pyrolyzed in air to form Si/MgO composites. The subsequent key acid etching step removes the MgO phase, leaving behind a porous silicon skeleton. Finally, a carbon coating process is carried out to produce p-Si@C composite material (Figure 8b–f). Structural characterization confirmed that silicon nanoparticles were uniformly coated in an amorphous carbon layer approximately 5 to 8 nm thick (Figure 8e,f). Compared with exposed porous silicon, the p-Si@C anode exhibits superior long-term cycling stability and maintains significant capacity at 1 A g^−1^ after 500 cycles (Figure 8g).

Etch-oriented strategies can efficiently construct mechanically robust, three-dimensional interconnected porous networks, promoting ion transport and electrolyte wetting. Its significant advantage lies in the abundance and low cost of raw materials (such as metallurgical silicon alloys), which makes this route particularly promising for large-scale production. However, there are still several challenges, including precisely controlling the uniformity and depth of etching to achieve a consistent pore structure, as well as addressing environmental and safety issues when using strongly acidic or corrosive etchants.

### 2.3. Deposition and Assembly

There are two main technical paths for deposition and assembly strategies: one is to directly construct porous silicon-based electrodes through gas-phase reactions, and the another is to organize components through interfacial forces or solution-type interactions. Unlike templates or etching methods, which typically rely on sacrificial strategies, current methods are often designed to build or integrate active materials directly into conductive, porous overall frameworks through a single step or simplified process. This strategy includes chemical vapor deposition (CVD), electrospinning, and a variety of solution-based assembly or printing methods, all of which offer unique advantages for making adhesive-free, integrated electrodes and electrodes with close electrical contacts and adjustable porosity.

Upon careful observation, vapor deposition technology—primarily chemical vapor deposition (CVD)—is capable of uniformly coating nanoscale silicon onto three-dimensional conductive scaffolds. A common concept involves growing a silicon layer on a carbon nanotube (CNT) sponge or graphene foam. In this type of process, a uniform silicon layer is deposited on a pre-formed porous carbon network. The resulting composite silicon–carbon material retains the interconnected porosity of the carbon skeleton, promoting rapid electron transfer and electrolyte penetration, while the nanostructured silicon coating can adapt to volume changes to achieve high capacity. In addition, the direct bonding of the Si/C interface ensures the mechanical integrity and robustness of efficient charge transfer kinetics.

Assembly and direct manufacturing methods integrate silicon nanoparticles with conductive or polymer matrices to form independent porous structures. A widely studied method involves preparing flexible silicon/reduced graphene oxide (rGO) composite films through vacuum filtration and subsequent thermal reduction [61]. In this way, silicon nanoparticles and graphene oxide flakes are uniformly dispersed in the solution during the filtration and subsequent reduction process, while rGO chips are assembled into a continuous and mechanically robust network that wraps around silicon particles and forms a layered porous structure. This design eliminates non-active elements such as metal current collectors and polymer bonds, thereby improving the overall specific energy of the electrode. Another innovative strategy is to directly laser print silicon graphene composite films [62], patterning the precursor ink containing silicon and carbon sources and rapidly processing them with a laser to generate porous conductive electrodes in one step. This technology precisely controls the electrode structure at the macro scale while ensuring effective electrical interconnection at the micro scale.

The principal advantage of vapor deposition and assembly strategies lies in their ability to create seamless, integrated electrode architectures with optimized interfacial contact and minimal inert mass. They offer exceptional multi-scale control over composition, morphology, and porosity, enabling the rational design of efficient ion- and electron-transport networks. Furthermore, these methods are particularly suitable for producing flexible, lightweight, or structurally complex electrodes for advanced battery designs. However, these advantages are often offset by notable limitations: high process complexity, considerable energy and time input, reliance on specialized equipment, and challenges in scaling up while maintaining uniformity and high active-material loading. The economic feasibility of such sophisticated techniques for large-scale manufacturing remains a major hurdle, motivating ongoing research toward process simplification or integration with more scalable production steps.

### 2.4. Comparative Analysis of Porous Si Architectures: Failure Modes, Mechanical Stability, and Design Trade-Offs

The electrochemical performance summarized in Table 1 is directly derived from the unique microstructure and configuration characteristics of various porous silicon structures. Every ingenious structural design, whether it is an egg yolk–eggshell structure, gradient pore structure, or three-dimensional interconnected network structure, has unique advantages, and there are also potential risks of structural failure and performance degradation. The core key to achieving rational design lies in a deep understanding of these potential “structure–performance” correlations and inherent material trade-offs, as shown in Table 2. However, before delving into the data in Table 1, a core question needs to be clarified: Do the various data listed in the table have direct comparability? Upon closer examination of testing conditions, it is not difficult to find that there are significant differences in active material loading, current density, and even voltage window among different studies, which greatly reduces the reference value of horizontal comparison solely based on capacity values. To this end, we made every effort to supplement key test parameters (such as the loading) in the table, providing an important background basis for readers to analyze the data.

Among them, the influence of the loading amount on the active material surface (mg cm^−2^) is particularly critical. A commonly overlooked fact is that extremely low surface loading of active materials (such as <1 mg cm^−2^) can result in artificially inflated performance due to reduced absolute expansion stress of the electrode, shortened ion transport pathways, and artificially increased high specific capacity and volumetric capacity values. On the other hand, there is a relative lack of systematic performance evaluation in existing literature for high-surface-loading conditions (>3 mg cm^−2^) that meet the requirements of commercial applications. This scarcity, itself, highlights the huge gap between academic exploration and industrial demand.

Apart from the loading, the “black box” of the test protocol has remained largely unopened. Electrode density/porosity, specific capacity values, electrolyte composition and quantity, and even seemingly minor voltage-window adjustments can significantly alter capacity data and degradation mechanisms. Although a unified benchmark test (such as normalizing the data to a volume capacity or area capacity that matches the anode) is ideal, the lack of original data often makes this “post hoc normalization” impractical. Therefore, the information provided in Table 1 is more of a “transparency statement”, reminding readers that these performance data must be viewed from the perspective of their specific context. Future research should give priority to reporting volumetric capacity, electrode density, detailed cycling protocols, and full battery performance data. Only by establishing a comparable foundation can the structural discussion evolve from the debate of “which is better” to a rational design of “which is more suitable for a specific application scenario”.

Returning to the structure itself, the yolk–shell design accommodates expansion through a dedicated internal void space, isolates the active core, and promotes the formation of a stable SEI on the surface of the inner shell. However, its drawbacks are equally evident. Complex multi-step syntheses often result in low tap densities, limiting the volumetric energy density. In addition, the limited point contact between the “yolk” and the “shell” may become a bottleneck when electron transmission is high. The gradient porous structure takes a different path, achieving a delicate balance between mechanical strength and ion transport by progressively relieving stress through a continually changing porosity from the core to the surface. However, precise control of this complex porosity gradient remains an ongoing challenge in the field of synthetic chemistry. In contrast, three-dimensional interconnected networks based on alloy precursor etching provide dual continuous pathways for electrons and ions, often leading to superior mechanical robustness and rate capability. However, the intrinsic high specific surface area of this design also exacerbates the loss of initial lithium, so complex interfacial engineering (such as laminated carbon coating) is required to achieve SEI stabilization.

Therefore, choosing a specific porous architecture is essentially a deliberate trade-off. Should we prioritize long-term cycling stability or extreme fast charging capability? Is the goal to pursue maximum volumetric energy density or process scalability? There is no universally perfect answer in the world—only optimal solutions that are suitable for specific application scenarios. By correlating the performance data in Table 1 with the mechanistic understanding revealed in Table 2, a clearer technical roadmap can be developed for the research and development of the next generation of high-performance silicon-based anodes.

### 2.5. Assessment of Synthesis Routes from an Industrial Scalability Perspective

From an industrial scaling perspective, making porous silicon for batteries requires a careful balance of cost, process complexity, and safety. A comparison of the three typical processes in terms of industrial scalability can be found in Table 3. The most practical method (magnesiothermic reduction) starts with cheap, plentiful raw materials. The big challenges here are controlling high heat and special atmospheres during production to get a consistent product. Other methods that use complex, expensive templates are much harder and more costly to scale up because they have many steps, and it is difficult to keep the quality the same in large batches. Chemical-etching processes are simpler but create a major barrier because they involve dangerous acids, demanding heavy investment in safety systems and waste treatment. In short, to scale up successfully, the focus must be on simplifying the core chemical process; carefully managing the production environment; and finding safe, cost-effective ways to build the right material structure.

### 2.6. Critical Challenges of Porous Si

Porous silicon is designed to manage its large volume changes during battery use, making it a promising but difficult material for high-capacity batteries. Its tiny porous structure helps prevent breaking but also causes three main, linked problems that block its commercial use.

The first challenges are low tap density and poor volumetric capacity. The intentional holes in porous silicon make it light and airy, leading to thick, loosely packed electrodes. As a result, while it stores a lot of energy by weight, the battery stores much less energy per unit of volume. For devices like phones and electric cars, where space is limited, this is a major weakness. The key is to balance having enough internal holes to handle swelling with making the particles dense enough to pack tightly. The second challenge is an unstable SEI on high-surface-area structures. A large area causes too many electrolytes to break down continuously, forming a thick, unstable SEI layer. This permanently uses up lithium and electrolytes, causing poor initial efficiency and fast energy loss. Additionally, the silicon’s repeated swelling and shrinking cracks this weak film, exposing new surfaces and continuing harmful side reactions. Creating a stable, thin SEI on this constantly changing, vast surface is a top challenge. The third challenge is the interconnected challenge. The high surface area that hurts stability is the same feature that reduces density. The growing SEI can also block the pores, making things worse. Therefore, the improvement of porous silicon anodes requires a complete plan that works on porosity, protects the surface, and increases electrode density all at once. Solving this three-part puzzle is key to using the material’s full potential in real batteries.

To address the critical challenges of porous silicon anodes, a multi-faceted improvement strategy is essential. To combat low tap density and poor volumetric capacity, structural optimization is key. This involves designing hierarchical or bimodal porous architectures and creating dense secondary particles. For the unstable SEI issue caused by a large surface area, surface and interface engineering is crucial. Effective approaches include constructing conformal artificial SEI layers, applying conductive coatings, and employing electrolyte additives that foster a stable, LiF-rich interface. Ultimately, the most promising solutions are integrated designs, such as yolk–shell or pomegranate-like structures. These smart shapes combine an internal porous silicon core to accommodate expansion with a protective, conductive outer shell, simultaneously enhancing density, stabilizing the interface, and improving overall electrochemical performance.

## 3. Advanced In Situ/Operando Characterizations of Si-Based Anodes

### 3.1. In Situ X-Ray Diffraction

In situ X-ray diffraction (XRD) is an advanced characterization analysis technique that can study the evolution of the crystal structure of materials in real time under battery operating conditions and is especially suitable for analyzing the electrochemical behavior of electrodes and the lithium storage reaction mechanism in energy storage systems. This technology is capable of dynamically keeping an eye on the alterations in the crystal structure, as well as phase transitions of materials when electrochemical processes are taking place. It provides essential experimental evidence that can help in the explanation of the mechanism through which materials change dynamically as the process of running a battery takes place. To date, the concept of in situ XRD technology has successfully been utilized in support of a range of research interests. As an example, it has been used to monitor the phase change of silicon-based anodes under low temperatures in the process of lithiation [81]. It has also been utilized to explore the intrinsic connection between the electrochemical performance of electrodes and their pre-lithiation degree [82]. Furthermore, it has been used to study the relationship between the changes in the thickness of electrodes and their electrochemical potential during cycling. These diverse application scenarios fully demonstrate the unique advantages and multifunctionality of in situ XRD technology in overcoming key challenges in the development of silicon-based anodes [81,82,83,84,85,86,87].

**Figure 9:**
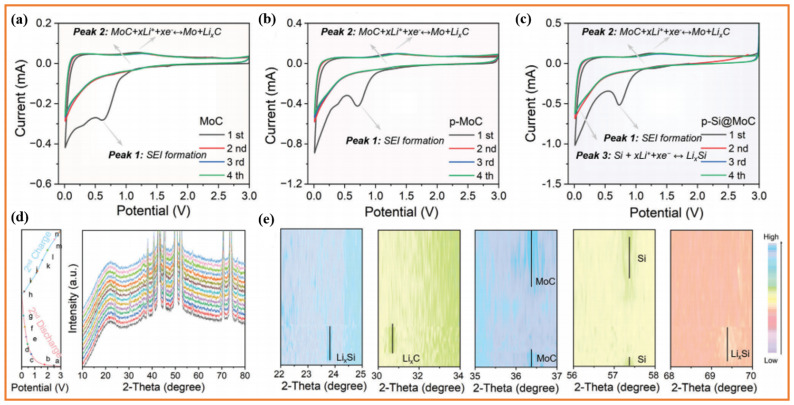
(**a**–**c**) CV profiles of MoC, p-MoC, and p-Si@MoC electrodes scanned between 0.01 and 3.0 V at 0.1 mV s^−1^. (**d**) In situ XRD patterns and corresponding galvanostatic profiles of p-Si@MoC at various voltages (“a–n” represents the sampling points). (**e**) Contour plots of the p-Si@MoC composite. Reproduced from [34], with permission from Wiley.

Chen et al. explored porous materials using in situ XRD to investigate the lithium storage mechanism of a p-Si@MoC anode [34]. The cyclic voltammetry (CV) curves shown in Figure 9a–c indicate clear redox peaks around 1.2 V (reduction) and 1.45 V (oxidation), which the authors attribute to reversible intercalation/decrystallization of the MoC matrix. Supplementary in situ XRD analysis (Figure 9d,e) provides structural insights: the diffraction peaks at 23.7° and 69.3° appearing during discharge, are grouped together as a formation of crystalline Li_x_Si, while the reappearance of the peak at 57.4° charging indicates regeneration of crystalline Si, confirming a reversible (de-)alloying process. In addition, the change in the intensity of the main MoC peak (~36.4°) and the simultaneous appearance/disappearance of the 30.7° peak during the cycle were interpreted as evidence of a conductive host-endoreversible conversion reaction.

Although this type of multimodal analysis has constructed a complete mechanism system, several key aspects still deserve further scrutiny and highlight the inherent analytical difficulties of this analysis method. Firstly, in the study, the diffraction characteristics at 30.7° were attributed to the crystalline MoC phase (Mo/Li_2_C), and the determination was largely based on the temporal correlation between the diffraction signal and the 1.2 V characteristic peak of the cyclic voltammetry (CV) curve. The signal source of the 1.2 V characteristic peak is still uncertain, and it may also originate from other crystalline substances, such as lithium molybdenum ternary compounds, or by-products produced by electrolyte decomposition. Secondly, to accurately determine the electronic configuration and atomic nature of the phase, other characterization techniques, such as in situ X-ray absorption spectroscopy (XAS) or high-resolution transmission electron microscopy (HRTEM) analysis, combined with selected area electron diffraction (SAED), are needed to detect the local atomic coordination environment and crystallographic characteristics of the material. Thirdly, the diffraction peaks of crystalline silicon characteristics observed after the recrystallization process also raise a core microstructural question: Does the silicon-based component recover to a single crystalline state after phase transition, or does it crack due to volume strain and form a polycrystalline or nanocrystalline network structure? Due to the fact that the signal from in situ XRD is a bulk-averaged result, it is not possible to distinguish between these two microstructural differences, which have a critical impact on the long-term mechanical integrity of silicon-based anodes and the lithium-ion transport pathway. Conducting in situ transmission electron microscopy (TEM) studies on similar composite structures will help to intuitively analyze the law of microstructural evolution.

In a wider range of silicon anode research, it has been clearly observed that reversible crystalline silicon phases and Li_x_Si phases are relatively rare, as amorphous phase transitions typically dominate. This phenomenon may be attributed to the unique architectural features of the studied system, particularly the low silicon content and its atomic dispersion, as well as strain fitness constraints within the porous MoC host. Therefore, the reported transition path from crystal to crystal may represent a specific situation of porous silicon-based anodes rather than a general mechanism. These mechanism-specific conclusions should be cautiously extrapolated to systems with high silicon loading or different host matrices. In summary, Chen et al.’s work demonstrates the value of combining in situ XRD with electrochemical measurements to propose detailed reaction pathways. Their explanation of the co-storage mechanism between silicon dopants and the MoC matrix is convincing, and the relevant data support is also sufficient. The research case is also able to teach us that, though there may be enough evidence collected through experimentation in order to reach the available conclusions, we still must maintain an open mind to other possible hypotheses. Further direct characterization studies are needed to improve the reliability of relevant mechanistic models due to the chemical nature of intermediate phases and the nanoscale morphological evolution of silicon-based materials. Therefore, true critical testing involves not only questioning specific explanations but also clearly defining the boundaries of their effectiveness.

Though in situ XRD is a powerful tool for tracking crystal phase evolution, its application to silicon-based anodes still has limitations, and data interpretation must take into account the insensitivity of this technique to amorphous phases or phases with low degrees of crystallinity. Key processes in silicon anodes, such as the formation of amorphous Li-Si alloys, the growth of solid–electrolyte interfaces (SEIs), and the irreversible amorphization of silicon during cycling, are not directly detectable, leading to an incomplete understanding of the reaction mechanism. Second, the signal of the electrode material may be obscured by background diffraction or absorption of battery elements, including transparent X-ray windows (e.g., Be, Kapton) and other internal components (current collector, separator). This interference is particularly severe in the early stages of SEI formation or when the silicon load is low. Third, traditional laboratory-scale in situ XRD often suffers from limited spatiotemporal resolution: spatial averaging of large electrode areas may mask local heterogeneous reactions, and slower data acquisition rates may fail to capture rapidly evolving transient phases under rapid electricity. Finally, the common XRD method provides depth-averaged information and cannot easily distinguish between surface-dominated processes (such as SEI evolution) and volume-centric structural changes—a key distinction in understanding degradation gradients within the electrode.

### 3.2. In Situ Transmission Electron Microscopy

In situ transmission electron microscopy (TEM) is an advanced characterization technique that integrates external stimuli such as thermal, electrical, magnetic, and chemical excitations based on the high spatial and energy resolution of traditional transmission electron microscopy. To date, this technology has been applied to real-time observation and recording of volume changes in silicon-based materials during petrochemical and dissolution processes [88,89,90,91,92,93,94,95,96,97,98,99,100,101]. Researchers have successfully applied in situ TEM to study various phenomena, including the real-time dynamic responses of materials to external stimuli [88], validation of zero-strain characteristics, and elucidation of atomic-scale storage mechanisms. Specifically, it has played an important role in studying the volume expansion mechanism of nano-silicon anodes, the critical role of the electrolyte environment, and the fundamental principles of atomic-level energy storage [89,90,91].

**Figure 10:**
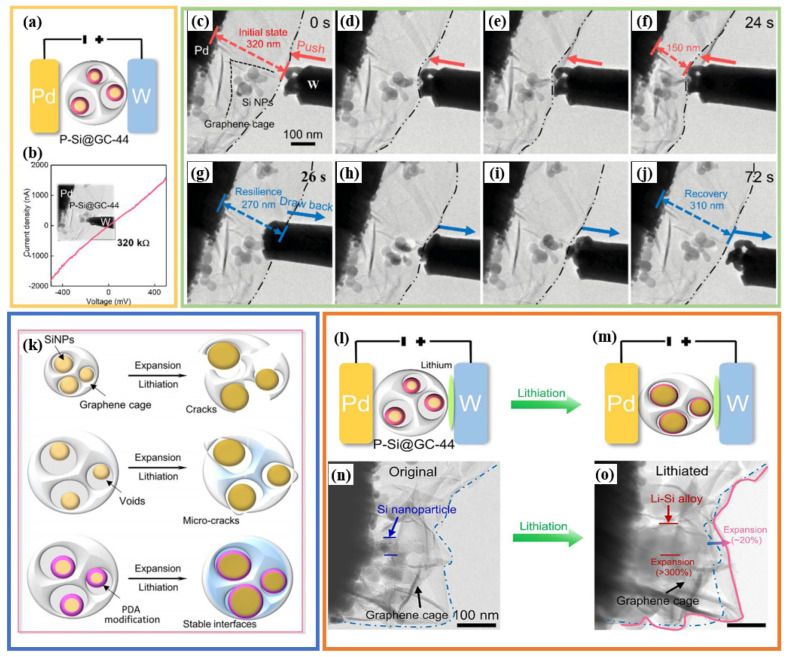
(**a**) Diagram of the electrical circuit for current–voltage measurements. (**b**) In situ TEM image and I-V curve of the carbon cage. (**c**–**j**) In situ TEM compression test of P-Si@GC-44. (**k**) Schematic of interface-strengthening strategy for thick Si anodes. In situ TEM observation of lithiation. (**l**,**m**) Schematics of the operando cell. (**n**,**o**) Lithiation and expansion of P-Si@GC-44 (the blue lines and arrows indicate the boundaries of the original materials, while the red lines and arrows point to the boundaries of the Lithiated materials). Reproduced from [102], with permission from Elsevier.

Through in situ TEM (Figure 10), Han and his colleagues captured the mechanical and electrochemical performance of graphene-encapsulated silicon nanoparticles (SiNPs) in real time [102]. Their experimental setup is very ingenious: they constructed a nanoscale “battery” inside the microscope, using a tungsten head as a lithium source to contact dopamine-coated and graphene-coated silicon particles (Figure 10a). This structural design system reveals to us many precious core principles. The results of the electrical measurements suggest that the resistance of the contact of the composite material is extremely low (only 320 kΩ, Figure 10b), which proves that the carbon network is able to achieve efficient and unrestricted transfer of electrons. The mechanical indentation test (Figure 10c–j) brings out a clear picture of the excellent elastic characteristics of graphene cages—they can be compressed by approximately 80% and can still recover close to 100% of the lost pressure when the pressure is released. The most novel outcome of the observation is the lithiation expansion process of silicon itself (see Figure 10n,o). The silicon core in the process has its volume increased by more than 300%, whereas a confined graphene cage under the influence of enormous mechanical stress never loses its integrity. The outer volume of the overall composite material only increases a bit—by approximately 20%. These images present conclusive and straightforward experimental evidence and exhaustively prove that high-toughness carbon skeleton models can completely package and hold back silicon-based active materials, thereby inhibiting their fragmentation and destruction during the cycling procedure.

To correctly analyze these research results, we should not underestimate the characteristics of the characterization research methods and should also provide a demonstration of the scope of the relevant conclusions. Firstly, the testing environment of TEM is far from the actual working conditions of the battery, making it impossible to fully replicate the complex conditions of the real battery system. The world inside a TEM instrument is a far cry from a real battery. Complex and continuous processes in liquid electrolytes, such as the continuous evolution of the solid–electrolyte interval (SEI), electrolyte penetration, and associated parasitic reactions, cannot be truly replicated in a high vacuum. Therefore, the mechanical toughness exhibited under these idealized short-term conditions may not fully predict the endurance of real batteries during medium- and long-term cycles, as electrochemical and mechanical stresses interact in complex ways.

Secondly, while dopamine (PDA) modification is, indeed, effective in improving the adhesion between diatomite particles and graphene, can this bond withstand hundreds of cycles of testing? Will this organic interfacial layer degrade progressively under repeated mechanical penalties of silicon expansion/contraction or as a result of a chemical attack on the electrolyte? A single short-term, in situ TEM experiment cannot answer the key question about long-term interface stability.

Thirdly, the minimal expansion of graphene cages in a single stone event is impressive, but it also begs another question. What about the fatigue problem? Periodically large internal stresses applied by the silicon core to the carbon cage may gradually introduce defects, cause sandwich sliding, or ultimately lead to fatigue failure—slow-accumulating damage mechanisms that cannot be captured by one-time experiments. Therefore, the “superior mechanical flexibility and stability” shown here should be understood more as strong proof of the initial cycle than a as guarantee of unlimited performance.

Finally, there is an urgent need to decisively investigate the inherent problem of intrinsic distortions in TEM technology, which is an in situ method. One important thing that is always underestimated is the possibility of methodological artifacts upon the carrying out of in situ TEM experiments. If we want to use this technology to analyze the mechanism of a silicon-based anode and integrate it into the electrode design system, we must face and solve a series of fundamental compromises that exist in the experimental process. At the same time, the high-energy electron beam used for detection in the experiment will also cause certain damage to the observed object, which will have a strong interference effect on the sample and may even change the intrinsic state of the material, thereby affecting the accurate analysis of the sample. It can initiate the radiodecomposition of electrolytes, catalyze and accelerate side reactions, induce local heating, and cause direct radiation damage to key components such as crystalline silicon and the solid–electrolyte interface (SEI). Therefore, the captured dynamic process may represent a system whose behavior has already been observed to change. Furthermore, the premise of electronic transparent samples, usually achieved in the form of nanowires or ultrathin films on dedicated chips, has greatly simplified the electrode structure. These model systems essentially lack the three-dimensional microstructure, hierarchical porosity and practical mechanical constraints specific to practical composite electrodes. Therefore, the deformation and fracture mechanisms elucidated under such idealized low-dimensional constraints may not be reliably transformed into the format of larger, higher-mass loads. Although in situ liquid battery TEM supports research in liquid electrolyte environments, it requires strict compromises in the application voltage window and electrolyte formulation to maintain battery integrity and reduce gas generation. The risk of these deviations from the standard electrochemical testing protocol is that they may guide the reaction to an atypical path, which may lead to mechanistic insights of limited practical significance. In fact, the core issue is that extracting quantitative data such as strain, thickness, or composition distribution from image sequences of dynamic transmission electron microscopy is particularly difficult. Developing reliable explanations is never easy. Most of them rely on complementary computational simulations and meticulous calibration to decompose the information behind complex image contrast. This inherently subjective analysis process also indicates that those attractive conclusions drawn from intuitive observations must undergo a rigorous validation and standardization process in order to be valid.

### 3.3. In Situ/Operando Raman Spectroscopy

In situ/operando Raman spectroscopy is an advanced analytical technique that can monitor the structural and compositional changes of materials in real time under active conditions or in specific dynamic processes. In the field of energy storage, in situ Raman spectroscopy has been widely employed to investigate phenomena such as strain evolution in carbon nanotubes during lithiation and delithiation [81].

**Figure 11:**
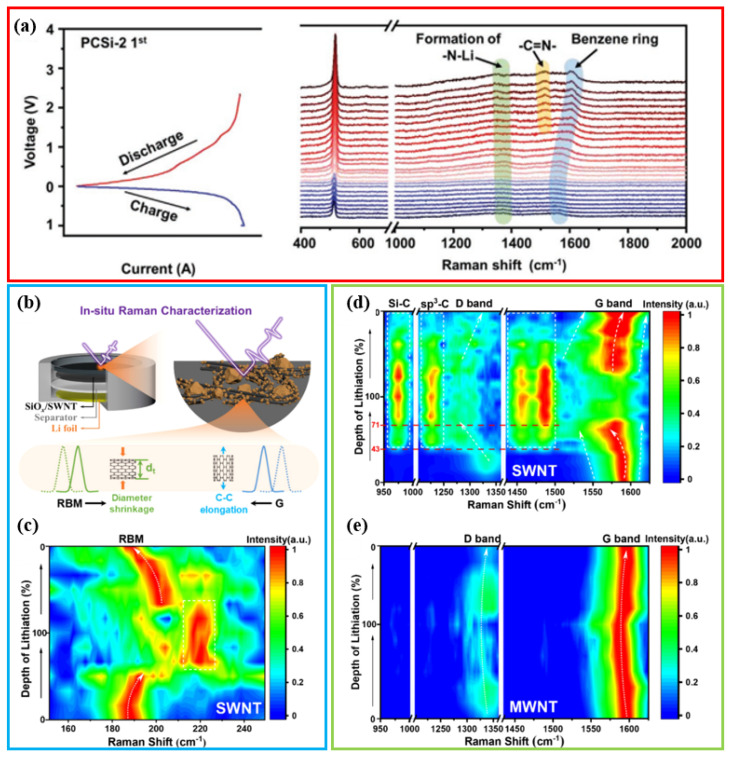
(**a**) Operando Raman response of PCSi-2 under CV conditions at 0.5 mV s^−1^. Reproduced from [59], with permission from Wiley. (**b**) Monitoring strain evolution via operando Raman spectroscopy: linking band shifts to structural changes in SWNTs. (**c**,**d**) Evolution of RBM and G/D band positions from operando Raman spectra during initial cycling of the SiO_x_|SWNT anode (the arrows indicate the changing trend of the positions of Raman peaks). (**e**) Evolution of G and D band positions in operando Raman spectra during the initial cycling of SiO_x_|MWNT. Reproduced from [103], with permission from American Chemical Society.

In a study by Wang et al., in situ Raman spectroscopy was used to analyze the lithium storage mechanism of a PCSi-2 anode [59]. The experiment revealed that during the delithiation process, lithium ions are preferentially removed from the benzene ring structure (when the voltage is <1 V). Subsequently, when the voltage rises above 1 V, the lithium in the -C-N-Li structure is removed and restored to the -C=N- structure (Figure 11a). The authors proposed that the residual lithium in PHATN forms a “molecular buffer” to accommodate the volumetric expansion of silicon and relieve mechanical stress. While this explanation is consistent with the observed evolution of the Raman peak, it is still predominantly phenomenological. Studies have not fully clarified whether the buffer effect is mainly due to the conformational changes of the polymer itself or the synergistic contribution of the underlying carbon interlayer and the binder-mediated hydrogen bond. Moreover, the long-term structural reversibility of PHATN under long, deep cycles has not been thoroughly verified after hundreds of cycles.

In contrast, Wang et al. focused on the role of single-walled carbon nanotubes (SWNTs) in SiO-based anodes [103]. Using operando Raman spectroscopy, the authors observed that lithiation-induced expansion of SiO exerts internal pressure on SWNTs, leading to a pronounced downshift of the G-band (Figure 11b–e), interpreted as tensile strain. Once the strain exceeded ≈14%, new Raman peaks associated with sp^3^-hybridized carbon and Si–C bonds emerged. This was attributed to a strain-driven interfacial reaction that stabilizes fragmented Si/SiO clusters and promotes the in situ reconstruction of a conductive electrode network. Although the proposed chemo-mechanical coupling mechanism is innovative, attributing the entire Raman shift solely to mechanical strain, without ruling out contributions from lithium intercalation into SWNTs or interfacial phase evolution, it may represent an oversimplification. The critical strain threshold (14%) and its causal link to Si–C bond formation, while supported by DFT modeling, would benefit from more direct experimental validation, such as in situ atomic-scale imaging or strain-controlled experiments.

Despite these interpretative challenges, both studies effectively demonstrate the ability of operando Raman spectroscopy to capture dynamic structural and interfacial changes in operating battery electrodes. Collectively, these works underscore the value of in situ spectroscopic tools in probing electrode dynamics while also highlighting the need for further multi-technique and mechanistic studies to fully unravel the complex interfacial processes in silicon-based anodes.

Operando Raman spectroscopy serves as a highly sensitive probe for detecting local chemical bonding and lattice strain in electrode materials. However, its application to practical Si-based anodes is subject to several inherent limitations that must be considered during data interpretation. First, laser-induced heating from the focused beam can alter local reaction kinetics, induce phase transformations, degrade sensitive components such as the SEI layer, or even trigger photochemical side reactions, thereby perturbing the electrochemical process under study. Second, the technique is inherently surface-sensitive, with a penetration depth typically on the order of micrometers. Consequently, Raman signals predominantly arise from near-surface regions or transparent electrolytes, while critical processes occurring within the bulk of thick composite electrodes may remain undetected. Third, fluorescence and the broad spectral background from carbon additives, binders, as well as electrolyte decomposition products, can overwhelm the weaker Raman features, especially during early cycles, when interfacial reactions are most active. Finally, although Raman peak shifts correlate with mechanical strain, deriving quantitative stress values remains challenging. This conversion requires accurate piezo-spectroscopic coefficients, which are difficult to obtain for heterogeneous, multiphase, Si-based composites, rendering strain analysis largely semi-quantitative in practice.

### 3.4. In Situ Electrochemical Impedance Spectroscopy

In situ EIS is an advanced analytical technology that can monitor the impedance characteristics of electrochemical systems in real time during operation or dynamic processes. Unlike conventional electrochemical impedance spectroscopy, in situ EIS allows for continuous measurement of electrode–electrolyte interface characteristics, such as battery charge–discharge cycles or electrocatalytic reactions, without interrupting system operation [104,105].

A critical assessment of the underlying mechanisms is essential. While factors such as SEI stabilization and improved physical contact within the electrode can contribute to reduced impedance, these effects are common to many composite systems and do not adequately explain the pronounced contrast between SWNT- and MWNT-based anodes. More specific insight is provided by converging evidence from complementary in situ techniques. For instance, the combination of operando Raman spectroscopy with density functional theory (DFT) calculations [103] has revealed that during lithiation, the volume expansion of SiO_x_ induces significant tensile strain in neighboring SWNTs. This strain, particularly at defect sites, promotes the formation of covalent Si–C bonds between SWNTs and lithiated silicon clusters. The establishment of such direct chemical linkages is understood to create highly efficient electron transfer pathways, thereby substantially lowering the interfacial charge-transfer resistance.

**Figure 12:**
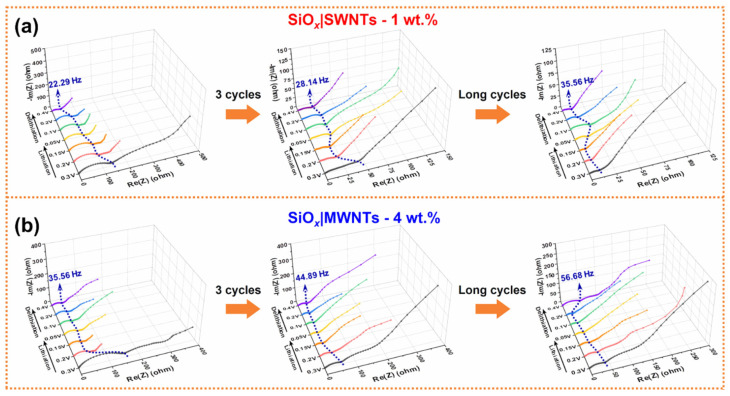
Evolution of in situ EIS Nyquist plots for (**a**) SiO_x_|SWNTs−1 wt% and (**b**) SiO_x_|MWNTs-4 wt% anodes during cycling. Reproduced from [103], with permission from American Chemical Society.

Furthermore, analysis of in situ EIS data (Figure 12) indicates a continuous improvement in Li^+^ diffusion kinetics throughout cycling, as reflected in the progressive decrease in Warburg impedance. This trend implies an ongoing structural reorganization within the electrode architecture. The volume changes of the active material are considered to mechanically engage the flexible SWNT network, promoting partial debundling of nanotubes, exposing fresh surfaces, and potentially opening additional ion transport channels. Enabled by the mechanical interplay between SWNTs and SiO_x_, this in situ architectural optimization complements the chemical bonding effect and contributes to sustained performance enhancement.

The interpretation of in situ EIS data, when integrated with other characterization methods, supports a dual mechanism for performance enhancement in SWNT-modified Si-based anodes: (1) strain-activated chemo-mechanical coupling that creates strong, conductive interfacial bonds and (2) cycling-induced structural reconstruction of the conductive network that facilitates ionic transport. This framework highlights that the role of SWNTs extends beyond passive conductivity, encompassing active participation in the electrochemical and mechanical evolution of the electrode.

In situ EIS is widely used for interfacial kinetics and ion transport probes in lithium-ion batteries, but silicon-based anodic EIS data present inherent challenges. A primary limitation is the model-dependent nature of data analysis. Physical parameters such as charge-transfer resistance, constant-phase element (CPE) values, and Li^+^ diffusion coefficients are extracted by fitting spectra to equivalent-circuit models. Model selection is often non-unique, and different choices can yield conflicting physical interpretations, especially for dynamically evolving interfaces like the growing/reconstructing SEI. Furthermore, overlapping electrochemical processes complicate spectral deconvolution. Charge transfer, SEI evolution, and Li^+^ diffusion through various phases frequently occur on similar time scales, leading to convoluted features in Nyquist plots that are difficult to disentangle. The impedance response is also sensitive to artifacts from cell geometry and measurement setup. Variations in cell configuration, current-collector morphology, electrode alignment, cable inductance, or inhomogeneous current distribution can distort high-frequency data, affecting estimates of bulk and interfacial resistances. Finally, EIS remains an indirect probe of interfacial changes. Although shifts in parameters signal interfacial evolution, the technique cannot identify the underlying chemical (e.g., SEI composition) or morphological (e.g., particle cracking, pore formation) alterations. Thus, correlative characterization is essential to link impedance variations to specific degradation mechanisms in Si-based anodes.

### 3.5. Unresolved Mechanitic Issues and Competing Perspectives

Although in situ characterization techniques have fundamentally changed our understanding of porous silicon anodes (Table 4), they have also revealed their complexity and occasionally fuzzy mechanisms. Due to the inherent limitations of in situ characterization techniques (Table 5), many fundamental issues remain unresolved, and there are conflicting explanations in the relevant literature.

What is the intrinsic nature of strain adaptation in porous networks? Pores are widely recognized to slow down volume expansion, but there is still intense debate on how this process occurs at the atomic and microstructural levels during continuous cycling. Does mechanical stress mainly induce reversible elastic deformation, irreversible plastic deformation, and pore collapse of porous skeletons, or is it a mixture of small pores merging into larger pores? In situ TEM can capture discrete snapshots of these processes, but it is still difficult to statistically quantify such phenomena over hundreds of cycles in actual composite electrodes. In addition, to date, the optimal aperture and interconnectivity for balancing long-term strain adaptation and rapid lithium transport have not been clearly defined, and design schemes still rely mainly on experience rather than deterministic theoretical guidance.

The evolution behavior of the SEI is equally perplexing: Is it dynamic self-repairing or irreversible growth? The significance of stabilizing the SEI has been widely recognized, but a core unsolved mystery is whether a truly dynamic and self-healing SEI can be formed on the vast and continuously fractured porous silicon surface. Some studies based on in situ EIS data support impedance reduction and propose that electrolyte additives can promote the formation of such adaptive interfaces. In contrast, other studies based on postmordial analysis have shown that the growth of the SEI is mainly an accumulative and irreversible process that continuously consumes lithium and electrolytes. The core of the debate lies in whether the chemical composition and form of the SEI can be engineered to have sufficient mechanical flexibility to withstand particle crushing without continuous thickening. There are also disputes regarding the crystalline and amorphous pathways in the evolution of the Li_x_Si phase. In situ XRD continuously captures the disappearance of crystalline silicon during the condensation process, confirming the amorphous phenomenon. However, the Li_15_Si_4_ crystal phase generated during deep discharge and its reversibility are greatly affected by the system. Certain results are observed to present clear Li_15_Si_4_ diffraction peaks that are reversibly removable upon charging. Some researchers, particularly those interested in nano-restricted or composite systems, have observed that, even at low potentials, the whole reaction process is entirely amorphous. The apparent mismatch here is an important and crucial query—namely, is the Li_15_Si_4_ phase, which is accompanied by a significant degree of volumetric strain, harmful or not harmful to the work of the electrodes? Will this stage occur during actual electrochemical cycling? Is its formation connected to local current density, silicon particle size, or the electrode’s stress condition? Resolving these pivotal issues is essential for the scientific planning of the charging and discharging mechanism in lithium-ion systems and the creation of high-performance silicon-based anodes.

Does the interfacial interaction between conductive additives mainly depend on chemical bonding or mechanical contact? The appearance of silicon–carbon bonds within single-walled carbon nanotube (SWNT) composites offers rather convincing proof for interfacial chemical coupling. Nevertheless, there is yet another important question that cannot be ignored: Is such covalent bonding absolutely necessary to attain outstanding electrochemical performance? Or could the close mechanical contact given by elastic conductive matrices (like specific polymers and graphene) be enough for charge transfer and structural stability? Up until now, researchers have not managed to effectively separate the respective contributions of chemical bonding interfaces, physical confinement effects, and electron transfer occurring under tunnel-stabilized gaps. This issue directly hinders the mechanism of optimizing the role of composite electrode design and imminently requires further probing to investigate the subject. To address these challenges, scholars must not just rely on more advanced in situ characterization methods of a fine analysis of silicon anode materials but should also consider multi-technique characterization to co-characterize the same sample as a way of obtaining mutual validation of the characterization data. We should admit that the ambiguity and controversies in the field of research of the mechanisms of interaction are not due to a lack of evolution of the scientific domain. Rather, the solution of these problems may offer definite guidelines to further research and contribute to the development of new discoveries in this field.

## 4. Performance in Full-Cell Configurations and Remaining Challenges

In order to effectively evaluate porous silicon anodes, it is necessary to transition from half-cell testing to full-cell measurements using high-capacity cathodes (such as NCM and NCA), operating under actual working conditions with limited lithium sources and total electrolytes. This change will expose many system-level issues that are easily overlooked in half-cell testing. This section combines typical results of all battery-related research to summarize the current core progress and unresolved bottleneck issues.

### 4.1. Progress in Full-Cell Integration: Strategic Designs and Demonstrated Performance

Through targeted material design and interface control, comprehensive performance of the battery can be achieved. The most representative study is by Wang et al. [103], who paired a silicon oxide anode doped with only a small amount of single-walled carbon nanotubes (1%) with a NCM811 cathode to assemble a full cell. The core key is to form a silicon carbon network structure inside the composite material that combines mechanical toughness and conductivity so that after 200 cycles at a rate of 0.2C, the capacity of this full battery can still be maintained at 80%. This also fully confirms the practical effect of in situ construction of covalent bonding interfaces in terms of improving cycling stability. In another more practical study, Li et al. [63] used honeycomb microporous silicon anodes. They contributed to the formation of a lithium-rich, stable solid–electrolyte interface (SEI) by customizing electrolyte formulations. After 500 rounds of full battery cycling at 0.5C using the NCM811 cathode, the anode could still maintain a stable capacity of 152.3 mAh g^−1^. This achievement also proves that well-designed micro-scale silicon-based structures have great potential to improve long-cycle durability.

### 4.2. Critical Bottlenecks Amplified in Full Cells

Despite these advances, the use of porous silicon in full cells has made many of its inherent problems more prominent. Firstly, there is the irreversible loss of lithium. The initial Coulombic efficiency of porous silicon is generally low, ranging from 75% to 85%, which leads to irreversible consumption of recyclable lithium provided only by the positive electrode in the battery, directly determining the energy density that the entire battery can achieve. Although some remedial methods have been used in relevant research, such as the use of lithium-rich cathodes [103] and optimization of SEI films [63], the development of stable, reliable, and scalable pre-lithiation technology remains a core issue that urgently needs to be addressed. Secondly, the issue of electrolyte depletion and mutual interference between cathodes and anodes is particularly challenging. Porous silicon has a large surface area and constantly changes dynamically, which greatly accelerates the decomposition of electrolytes. The entire battery is sealed, while the total amount of electrolytes is limited. This accelerated decomposition can easily cause the electrolytes to dry up, leading to rapid battery failure. More seriously, there may also be crosstalk between cathodes and anodes: at high operating voltages, transition-metal ions such as manganese and nickel will dissolve from NCM cathodes and flow to the anode surface of silicon, which not only causes more side reactions but also damages the stability of the SEI film—a fault problem that cannot be detected in half-cell testing. In addition, the dynamic variation of the silicon anode volume is also a major challenge in limited battery stack structures. The silicon anode will repeatedly expand and contract during the cycling process, but it can only be completed within a fixed battery volume, which can cause internal pressure fluctuations. After long-term cycling, such pressure fluctuation can cause the electrodes to slowly peel off and lose electrical contact and can even lead to physical deformation of the battery components—problems that do not occur in unconstrained half cells.

### 4.3. Pathways Toward Viable Integration

In order to promote the commercialization of future porous silicon anodes, researchers must prioritize indicators and strategies related to the entire battery system. Firstly, they should focus on the structural design of porous anodes while achieving high initial Coulombic efficiency, high volume density, and mechanical toughness to minimize system-level performance compromises. The second priority is to develop a dual-function electrolyte formula that can not only stabilize the interface of the high-voltage cathode but also stabilize the interface of the silicon anode, reducing the crosstalk problem between the electrodes. The third priority is to develop and adopt a full-cell testing plan that meets actual usage conditions, such as by limiting the amount of electrolytes, using actual negative and positive electrode capacity ratios (N/P ratio > 1.1) and electrolyte/capacity ratios (E/C ratio), and identifying the fault mechanism through long-term cycling. The fact that effectively converting excellent half-cell properties in the lab to commercial products is, in large part, a large system integration problem calls upon researchers to jointly refine the design of the anode, the matching of the cathode, electrolyte development and the overall battery architecture.

## 5. Conclusions and Outlook

This review provides a comprehensive analysis of the structural design of porous Si-based anodes and their applications in lithium-ion batteries while highlighting the pivotal role of advanced in situ characterization techniques in elucidating their electrochemical behavior. The related studies demonstrate that the design of porous architectures is a highly effective strategy for mitigating the volume expansion challenges inherent to Si anodes. Through different approaches, the design of synergistic silicon-based composites [107,108,109,110] has facilitated the ongoing development of diverse high-performance porous silicon-based anode materials, significantly improving their performance. Furthermore, the advancement of in situ characterization techniques—such as in situ XRD, TEM, Raman spectroscopy, and EIS—has greatly enhanced our understanding of the operational mechanisms of Si-based anodes. These techniques enable real-time monitoring of structural evolution, interfacial dynamics, and reaction characteristics, providing multi-faceted insights into the lithium storage and failure mechanisms of Si-based anodes.

Although considerable advances have been achieved in this domain, the methods of translating laboratory research discoveries into business-ready technologies still demand an objective and elaborated look at a set of issues, as opposed to the typical solution of just the one dilemma of volume augmentation. To close the gap between laboratory studies and mass production on an industrial scale, future research should not be based only on the investigation of previously discovered effects. It is up to researchers to pre-empt the establishment of other types of technological directions that are not only viable but that also have the ability to be industrialized. To this end, below, we present some general research directions, which, through rational structural design, interface engineering, scalable preparation, and novel characterization methods, we intend to transform the above serious issues to disruptive development opportunities.

Firstly, future studies must concentrate more on the creation of rational and sustainable structural designs for industrial production. Researchers must shift the focus of their efforts to developing materials that can be produced in large quantities and without compromise. To achieve this goal, easily accessible and inexpensive materials should be utilized for research, for example, the production of silicon and carbon materials derived from biomass [110]. Appropriate in situ characterization can reveal the real-time changes in materials during electrochemical processes, providing a basis for material mechanistic analysis and optimization design. For instance, by employing in situ Raman spectroscopy [103], it is possible to conduct auxiliary examinations of materials that can absorb stress, thereby enabling the determination of the main components of materials that generate significant stress and guiding the further optimization of anodes.

The second direction for future research is related to the control of dynamic interfaces and scalable pre-lithiation engineering. The method of establishing an adaptive interface system is an important research area in this field, which is more advanced than the traditional static surface coating method. It involves researchers developing dedicated polymer binders, as well as electrolyte additives, to build up a stable SEI film that is not merely thin and flexible but also self-healing [96]. Simultaneously, considerable attention should be paid to optimizing and enhancing scalable and reliable pre-lithiation methods. For example, with a pre-lithiation process that has been used to stabilize the anode material [111], it is possible to compensate for the loss of lithium in the first cycle of the battery, thereby allowing the entire battery to actively use its actual energy density.

The third approach is to utilize artificial intelligence, such as multi-scale simulation and machine learning, to accelerate the multi-scale design of porous structures and simultaneously overcome the inherent contradiction between porosity and tap density. Such efficient tools can be used to quickly filter out options to identify the best pore topology. By so doing, the fully mechanical strain-buffering system is brought to its utmost, and simultaneously, the loss of tap density is minimized [112,113].

Finally, in order to better understand the complex degradation process of porous silicon anodes, comprehensive characterization methods applicable under different conditions should be employed. On the one hand, although in situ characterization can play a very significant role in understanding the investigated mechanism, individual in situ characterization often only provides very limited information and cannot fully understand all the detailed changes and overall alterations experienced by the battery [114,115,116]. In this situation, it is an important future trend to simultaneously employ two or more in situ characterization techniques to gain a deeper understanding of the material’s change mechanism. On the other hand, some specific in situ environments need to be developed, such as extremely low- or high-temperature conditions, high-voltage conditions, and so on, to meet the requirements of future research on batteries for extreme conditions.

The commercialization of porous silicon anodes requires interdisciplinary collaboration across various fields. Only by comprehensively considering factors such as cost-effectiveness, the scalability of production, volumetric energy density, and service safety and integrating these considerations into fundamental material research can the significant application potential of porous silicon materials be transformed into the core supporting technology for the next generation of high-energy-density lithium-ion batteries.

## Figures and Tables

**Table 1:** Structural design and electrochemical properties of porous Si-based anode materials for LIBs.

Structure Category	Material	Initial Capacity (mAh g^−1^)	ICE (%)	Current Density (mA g^−1^)	Cycling Stability	Rate Performance	Areal Loading (mg cm^−2^)	Voltage Window (V)	Ref.
Yolk–Shell/Hollow	Mesoporous Si hollow cubes	~2800	~80	2000	850 mAh g^−1^ after 800 cycles	1050 mAh g^−1^ at 30 A g^−1^	0.5–1.0	0.01–1.0	[60]
3D Porous Network	Gradient porous Si (GP-Si)	~2500	76.5	2000	1059 mAh g^−1^ after 500 cycles	~1200 mAh g^−1^ at 10 A g^−1^	0.8–1.2	0.01–1.5	[31]
3D Porous Network	Interconnected p-Si@C network	~2300	~70	1000	>800 mAh g^−1^ after 500 cycles	Good retention at 2 A g^−1^	0.6–1.0	0.01–1.5	[32]
Doped Composite	Si-doped porous MoC (p-Si@MoC)	866	98.4	200	977 mAh g^−1^ after 250 cycles	893 mAh g^−1^ at 0.1 A g^−1^	~1.0	0.01–3.0	[34]
Nanocomposite	Si/rGO film	1727	71	200	1024 mAh g^−1^ after 200 cycles	680 mAh g^−1^ at 3 A g^−1^	0.5–1.0	0.01–1.5	[61]
3D Composite	3D graphene-Si matrix	~3000	~75	2500	~437 mAh g^−1^ after 4500 cycles	~500 mAh g^−1^ at 10 A g^−1^	~1.5	0.01–1.0	[62]
Porous Gradient	Honeycomb micro-sized Si	2409	90.07	~430 (0.9A/g)	74.21% after 200 cycles	78.0% SOC at 0.9 A g^−1^	1.0–1.5	0.01–1.0	[63]
Hierarchical	Coral-like p-Si@N-doped C	1388	76.93	1000	804 mAh g^−1^ after 300 cycles	1209 mAh g^−1^ at 4 A g^−1^	0.8–1.2	0.01–1.5	[64]
3D Skeletal	Skeletal porous Si	2300	65.8	100	1607 mAh g^−1^ after 200 cycles	1315 mAh g^−1^ at 3.6 A g^−1^	~1.0	0.01–1.0	[65]
Surface/Interface Engineered	Ah-Pr-SiO	~1620	99.4	1000	1435.8 mAh g^−1^ after 200 cycles (88.7%)	1031.2 mAh g^−1^ at 3 A g^−1^	~1.0	0.01–1.5	[66]
Core–Shell	SiNPs@hSEI-L	2615	83.5	2000	1044.7 mAh g^−1^ after 500 cycles	1439.8 mAh g^−1^ at 4 A g^−1^	N/A	0.01–1.5	[67]
Coating/Composite	B–Si NPs@ZNCN	826.8	54.6	400	723.4 mAh g^−1^ after 1000 cycles	410.3 mAh g^−1^ at 3000 mA g^−1^	N/A	0.01–1.5	[68]
Coating/Composite	B-3DCF/Si@C	~1288.5	75.2	400	1288.5 mAh g^−1^ after 600 cycles	988 mAh g^−1^ at 2000 mA g^−1^	~0.5	0.01–1.5	[69]
Core–Shell/Composite	Si@SiO_x_@BNCNT	~1700	N/A	200	1045 mAh g^−1^ after 700 cycles	620 mAh g^−1^ at 4000 mA g^−1^	N/A	0.01–1.5	[70]
Surface-Coated Composite	p-Si@g-C_3_N_4_	~2100	~60	1000	1252 mAh g^−1^ after 500 cycles	1259 mAh g^−1^ at 2 A g^−1^	~0.5–1.0	0.01–1.5	[71]
Thin Film/Amorphous HEA	Si_50_(AlMgGeSn)_12.5_ HEA thin film	2251	−	0.1 mA cm^−2^	94.6% after 50 cycles	1421 mAh g^−1^ at 0.5 mA cm-2	~0.2	0.01–1.5	[72]
Core–Shell/Gradient	Si@SiO_2_@C	~2323	83	2000	856 mAh g^−1^ after 1500 cycles	744 mAh g^−1^ at 5 A g^−1^	0.67–2.1	0.01–1.5	[73]
Yolk–Shell/Hollow	Si@Li_2_O@TiO_2_ nanosheet	2637	90.9	2000	~1300 mAh g^−1^ after 1150 cycles	>900 mAh g^−1^ at 20 A g^−1^	0.72	0.01–1.0	[74]
Surface-Coated	CS-decorated Si NPs	3197.2	92.2	~1600 (0.5C)	1621.3 mAh g^−1^ after 500 cycles	~2200 mAh g^−1^ at 10 C	~1.0	0.01–1.0	[75]
Yolk–Shell/Hollow	pSi@void@NMC	1861.5	77.8	200	1769.8 mAh g^−1^ after 300 cycles	Good retention up to 3 C	1.0–1.5	0.01–3.0	[76]
Micron-SiC Composite	Micro-sized SiC_x_	1455 (0.1C)	85.9	~291 (0.2C)	95.8% after 100 cycles	Good retention at 2C	~2.4	0.005–1.5	[77]
Self-Healing Binder Composite	Si/C with SHIR-A1 binder	~402 (0.1C)	−	~402 (1C)	83.3% after 300 cycles	~220 mAh g^−1^ at 2C	~1.52	0.001–1.5	[78]
Micron-Si Low-Temp Composite	Micro-sized Si (µSi) with MDFA/FEC	1302 (−40 °C)	−	100	786 mAh g^−1^ after 100 cycles (−40 °C)	1249 mAh g^−1^ at 0.1 A g^−1^ (−40 °C)	−	0.005–1.5	[79]
Core–Shell Composite	Gr@Si@C	1622	87.9	~220 (0.5C)	72.2% retention after 100 cycles	1240 mAh g^−1^ at 3C	~2.5	0.01–1.5	[80]
	CG-Gr@Si@C	~465–630	~91–92	~220 (0.5C)	78.3% retention after 100 cycles	High areal capacity 3.5 mAh cm^−2^	~4.2–11	0.01–1.5	

**Table 2:** Comparison of representative porous Si-based anode architectures: Synthesis, advantages, failure modes, and design trade-offs.

Structure	Synthesis Route	Key Advantages	Potential Failure Modes	Mechanical Stability Limits/Critical Factors	Design Trade-offs	Ref.
Yolk–Shell	Template-guided	Expansion buffer; stable inner SEI	Shell fracture; contact loss	Shell strength; void volume	Stability vs. low density	[34,60]
Porous Gradient Structure	Etching-directed	Stress gradation; balanced transport	Gradient inhomogeneity; pore collapse	Porosity gradient; core strength	Balanced performance vs. synthesis control	[31]
3D Interconnected Network	Etching-directed	Bicontinuous transport; robust structure	SEI overgrowth; high initial loss	Network connectivity; etching uniformity	Rate/life vs. interface engineering	[32,65]
Hollow Sphere	Template-guided	Large void; short ion path	Shell fracture; low packing	Shell strength & uniformity	High capacity vs. low density	[60,61]
Core–Shell	Vapor deposition, coating, or assembly	Core protection; enhanced conduction	Shell cracking; limited expansion	Shell porosity/flexibility; modulus match	Conductivity vs. fracture risk	[81]

**Table 3:** Scalability and challenges for different Si anode manufacturing routes.

Aspect	Magnesiothermic Reduction	MOF Templating	Chemical-Etching Route
**Scalability assessment**	High potential. Utilizes abundant, low-cost raw materials (e.g., silica) with a relatively simple and short process flow.	Low scalability currently. The synthesis involves complex, multi-step procedures that are difficult to control and reproduce uniformly in large, continuous batches.	Moderate to good potential. The solution-based, low-temperature process is inherently scalable, but handling large volumes of hazardous chemicals presents engineering challenges.
**Main cost barriers**	High energy consumption, particularly in traditional high-temperature (>650 °C) processes. Costs associated with maintaining an inert atmosphere.	Exceptionally high precursor costs (MOF materials, silicon precursors). Low overall yield and the need for precise process control further increase expenses.	Cost of chemicals. Economics heavily depend on effective recycling and hazardous waste treatment.
**Primary safety/process obstacles**	Managing the highly exothermic reaction and preventing particle sintering. Requires strict inert atmosphere control throughout the process.	Use of volatile and flammable organic solvents. Requires high-temperature treatments in controlled atmospheres. Difficult to ensure product uniformity at scale.	Extreme hazard from highly toxic and corrosive hydrofluoric acid (HF), demanding specialized equipment and rigorous safety protocols. Environmental handling of heavy metal waste.

**Table 4:** In situ characterization techniques for the detection of Si-based anode materials and associated research contents.

In Situ/Operando Characterization Technique	Research Direction	Research Contents	Ref.
In situ XRD	Structural evolution and lithium storage mechanism	Investigation of low-temperature phase evolution	[81]
		Relationship between electrodes and prelitigation	[83]
		Lithium storage mechanism	[34]
		Relationship between electrode thickness and electrode potential	[84]
In situ/operando Raman spectroscopy	Lithium storage mechanism and reaction characteristics	Demonstrate the formation of SEI (Solid—Electrolyte Interface)	[106]
		The strain of carbon nanotubes during lithiation and degradation processes	[81]
In situ TEM	Material properties and microscopic mechanisms	Investigation of the volume-expansion mechanism of nano-silicon anodes and the electrolyte environment	[89,90,91]
		Investigation of structure, phase transition and volume evolution during the lithiation process	[102]
		Verify the zero-strain characteristic of materials and the storage mechanism at the atomic scale	[88]
		Verify the mechanism and optimal point of stress-adaptive binders	[96]
		New reaction mechanism of active Si and Ge bilayers	[99]
		Storage mechanism at the atomic scale	[91]
In situ EIS	Solvation structure and interface evolution	Investigate the comprehensive impact of TMSB on the Li^+^ solvation structure	[105]
		Evolution process of the interfacial SEI film	[103]
		Impedance spectroscopy during the charge–discharge process	[104]

**Table 5:** In situ/operando characterization techniques for porous Si-based anodes: capabilities and limitations.

Technique	Core Capabilities/Probed Phenomena	Key Limitations for Si Anode Studies	Typical Spatial & Temporal Resolution
In situ XRD	Phase transitions (cryst. Si ↔ Li_x_Si)	Insensitive to amorphous phases (SEI, a-Li_x_Si)	Spatial: μm–mm (bulk) Temporal: Seconds to minutes
	Crystallographic evolution	Signal interference from cell components	
	Strain in crystalline phases	Poor spatial resolution (bulk averaging)	
		Slow for fast kinetic processes	
In situ TEM	Real-time morphological evolution	Electron-beam damage (SEI, electrolytes)	Spatial: Å–nm (atomic to nanoscale) Temporal: Milliseconds to seconds
	Visual observation of fracture/expansion	Non-representative thin samples	
	Interface dynamics at atomic scale	Constrained electrochemical window in liquid cells	
		Complex quantitative analysis of images	
In situ/operando Raman spectroscopy	Chemical bonding evolution (Si, C, SEI)	Laser-induced heating/photochemistry	Spatial: ~1 μm (diffraction limit) Temporal: Seconds
	In situ strain mapping in electrodes	Surface-sensitive (μm depth)	
	Phase identification of intermediates	Fluorescence interference	
		Semi-quantitative strain analysis	
In situ EIS	Interfacial kinetics evolution	Model-dependent interpretation	Spatial: N/A (macroscopic average) Temporal: Minutes per spectrum
	SEI growth & Li^+^ diffusion dynamics	Difficulty deconvoluting overlapping processes	
	State-of-health monitoring	Sensitive to cell geometry artifacts	
		Indirect probe (requires corroboration)	

## Data Availability

No new data were created or analyzed in this study. Data sharing is not applicable to this article.
